# Music-reading expertise alters visual spatial resolution for musical notation

**DOI:** 10.3758/s13423-012-0242-x

**Published:** 2012-03-30

**Authors:** Yetta Kwailing Wong, Isabel Gauthier

**Affiliations:** 1 Department of Psychology, University of Hong Kong, 604, Knowles Building, Pokfulam Road, Hong Kong, People’s Republic of China; 2 Vanderbilt University, Nashville, Tennessee USA

**Keywords:** Reading, Object recognition, Music cognition, Perceptual expertise, Crowding, Reading speed

## Abstract

Crowding occurs when the perception of a suprathreshold target is impaired by nearby distractors, reflecting a fundamental limitation on visual spatial resolution. It is likely that crowding limits music reading, as each musical note is crowded by adjacent notes and by the five-line staff, similar to word reading, in which letter recognition is reduced by crowding from adjacent letters. Here, we tested the hypothesis that, with extensive experience, music-reading experts have acquired visual skills such that they experience a smaller crowding effect, resulting in higher music-reading fluency. Experts experienced a smaller crowding effect than did novices, but only for musical stimuli, not for control stimuli (Landolt Cs). The magnitude of the crowding effect for musical stimuli could be predicted by individual fluency in music reading. Our results highlight the role of experience in crowding: Visual spatial resolution can be improved specifically for objects associated with perceptual expertise. Music-reading rates are likely limited by crowding, and our results are consistent with the idea that experience alleviates these limitations.

Musicians read music scores rapidly to transform visually complex musical notation into music. Music-reading experts can encode four-note music sequences three times faster than novices can (Wong & Gauthier, 2010a, b). What mechanisms contribute to this impressive perceptual performance for musical notes? Prior work has suggested that experts process music sequences holistically and automatically, with the amount of holistic processing being related to individual differences in music reading speed (Wong & Gauthier, 2010a). In this article, we explored another factor that may be involved in music-reading expertise—visual crowding.

*Crowding* refers to the disruption of visual processing by distractors located close to the target, and it is considered a fundamental limitation on visual spatial resolution (see the recent reviews in Levi, 2008; Pelli & Tillman, 2008). This limitation is particularly robust in the peripheral visual field, where an isolated object can be identified easily, yet recognition is disrupted once flankers (distractor objects) are added close to that object. Crowding occurs when the distance between the flankers and the target object is less than a critical distance, which is roughly half of the eccentricity of the target in the visual field (the *Bouma law*; Bouma, 1970; Pelli & Tillman, 2008). The causes of crowding remain controversial, with proposed theories ranging from the limitation of neural structures (e.g., large receptive fields or long-range horizontal connections in visual periphery; Levi, 2008; Levi & Waugh, 1994), to inappropriate featural integration of the targets and flankers within a spatial region (Pelli & Tillman, 2008), compulsory or preattentive averaging of target and flankers (Greenwood, Bex, & Dakin, 2009; Parkes, Lund, Angelucci, Solomon, & Morgan, 2001), and the insufficient resolution of spatial attention (He, Cavanagh, & Intriligator, 1996; Tripathy & Cavanagh, 2002).

Crowding is likely an important factor that limits the efficiency of music reading, as is the case with word reading. In word reading, the reading rate is thought to be limited by crowding, because letter identification is limited by crowding from adjacent letters (Levi, 2008; Pelli & Tillman, 2008; Pelli et al., 2007; Whitney & Levi, 2011). Similarly, fluency in music reading may be limited by crowding, as each musical note is crowded by adjacent notes and by the five-line staff. Can music-reading experience help experts alleviate crowding, such that they attain improved music-reading rates?

In the literature, the relationship between crowding and perceptual experience has not been addressed until recently. According to one theory, crowding is independent of object category, regardless of whether we are highly experienced with discriminating between objects in that category (e.g., letters or faces) or are not experienced (e.g., chairs or Gabor filters; Pelli & Tillman, 2008). Also, it is common to conduct crowding experiments with participants well practiced, or even pretrained, with the tasks, as well as with small sets of stimuli (e.g., Louie, Bressler, & Whitney, 2007; Martelli, Majaj, & Pelli, 2005; Petrov, Popple, & McKee, 2007; Tripathy & Cavanagh, 2002; Zhang, Zhang, Xue, Liu, & Yu, 2009) in which the possible influence of perceptual experience on crowding is largely ignored.

However, research has suggested that crowding can be affected by experience. For example, videogame players experience a smaller crowding effect than do nonplayers (Green & Bavelier, 2007). When numbers in a recognition experiment were flanked by Roman letters, performance decreased in the upper left visual field for native English speakers, but not for speakers of Asian languages (e.g., Japanese, Chinese or Korean; Williamson, Scolari, Jeong, Kim, & Awh, 2009). Crowding for upright face recognition is stronger when the flankers are upright rather than inverted faces (Louie et al., 2007); sensitivity to the orientation of faces is thought to be related to our perceptual experience with upright faces (Bukach, Gauthier, & Tarr, 2006). Crowding effects are smaller for letters than for symbols (Grainger, Tydgat, & Issele, 2010). More direct evidence for comes from training studies, in which recognition of a crowded letter can be improved after several hours of practice with the same task (Chung, 2007; Huckauf & Nazir, 2007) and in which the improvement generalizes to untrained spacing between targets and flankers (Chung, 2007), suggesting that perceptual training can alleviate crowding.

In this study, we tested whether music-reading expertise alleviates crowding. We measured crowding of musical stimuli by the staff lines and by flanking notes in music-reading experts and novices. Nonmusical stimuli were also used, to examine whether any expertise effects were specific to musical notation or instead resulted from a more general ability to resolve crowded shapes. If music-reading expertise can alleviate crowding in a domain-specific manner, experts should experience a smaller crowding effect than do novices for musical but not for control stimuli. In addition, we measured far and near acuity and contrast sensitivity for each individual, to test whether group differences in basic visual functions accounted for any expertise effect of crowding.

Our second goal was to clarify the relationship between crowding and reading rate. Recent work on word reading suggested that critical spacing for letter identification can be predicted by the critical spacing for reading (Pelli et al., 2007), and that reading rate is limited by the size of the “uncrowded window”—that is, the number of letters one can recognize in one fixation (Pelli & Tillman, 2008; see also Levi, 2008). However, explicit training on crowding has improved the ability to recognize a crowded letter without improving reading rate (Chung, 2007). Here, we measured individual perceptual fluency for music sequences as a measure of individual music-reading rates, which varied greatly among our participants (Wong & Gauthier, 2010a, b). If crowding limits reading rate, the amount of crowding experienced by each individual should be predicted by his or her perceptual fluency with musical notes.

## Method

### Participants

A group of 24 experts and 20 novices completed the experiment for cash payment. All of the participants reported their experience in music reading and rated their music-reading ability (1 = *do not read music at all*; 10 = *expert in music reading*). Their handedness was assessed by the Edinburgh Handedness Inventory (Oldfield, 1971). The experts included 12 females and 12 males (*M*_age_ = 22.9, *SD* = 6.2; 22 right-handed, 1 left-handed, 1 ambidextrous), with an average of 13.7 years of music-reading experience and a self-rated score of 9.08. The novices included 9 females and 11 males (*M*_age_ = 25.0, *SD* = 6.4; 19 right-handed, 1 left-handed), with 0.41 years of music-reading experience and a self-rated score of 1.35. All reported normal or corrected-to-normal vision and gave informed consent according to the guidelines of the institutional review board of Vanderbilt University. They were paid $12 per hour of behavioral testing.

### Stimuli and design

**Fig. 1:**
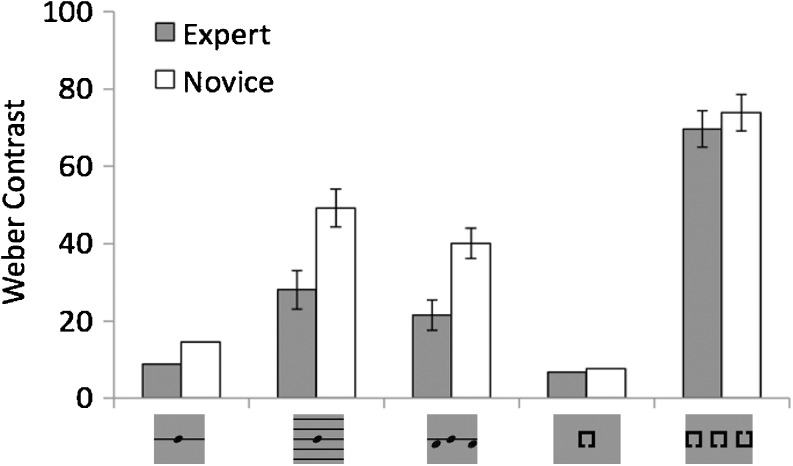
Contrast thresholds for all of the baseline or crowded conditions. Error bars plot the 95% confidence intervals (CIs) for the Group × Crowding results for each type of crowding. Error bars for the baseline conditions were omitted because they were associated with different sets of error bars in different ANOVAs

**Fig. 2:**
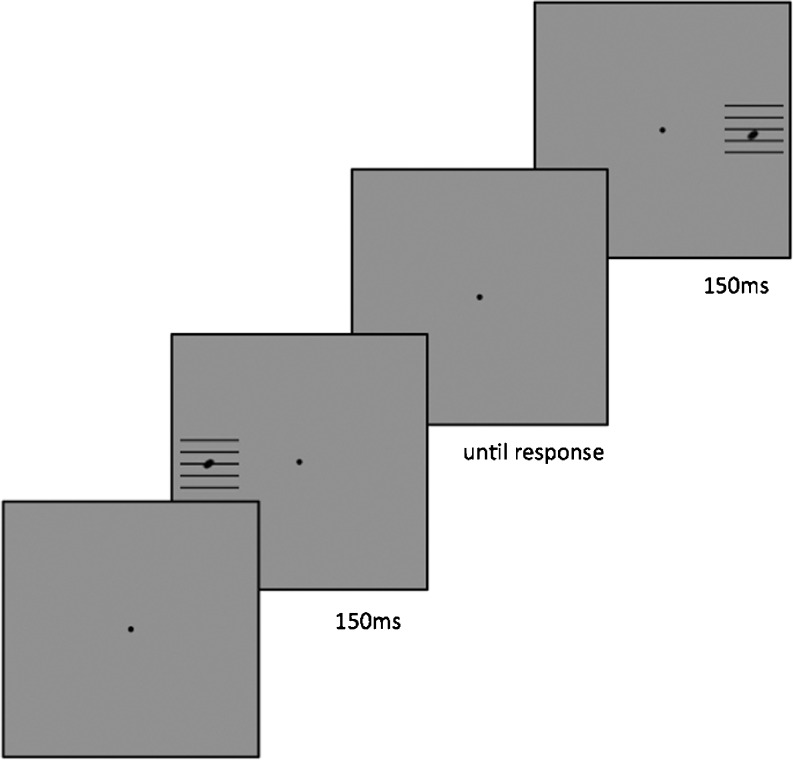
Experimental paradigm for the crowding task

The experiment was conducted on Mac Minis using MATLAB. The stimuli subtended about 1.3º × 1.3º of visual angle on a CRT monitor (28.2 cd/m^2^) in a dimly lit room. The baseline included a line and a dot, sometimes crowded with four additional staff lines or two flanking dots (see the *x*-axis in Fig. 1). Participants were asked to judge whether the (central) dot was on a line or on a space (Fig. 2). Landolt Cs were used as the nonmusical controls, for which participants judged whether the gap was at the top or the bottom (see Fig. 1). The target–flanker distances ranged from 0.22º to 0.43º for musical stimuli and 0.64º for Landolt Cs, well within the critical spacing for crowding (half of the eccentricity of the stimuli = 1.3º; Bouma, 1970). Each trial included a 500-ms central fixation, and then a 100-ms stimulus randomly presented at 2.6º to the left or right of fixation. The participants responded by keypress without a time limit.

Trials for each condition were blocked, with block order counterbalanced. Weber contrast thresholds for 75% accuracy were estimated four times, each with 40 trials, using QUEST. The contrasts of the target and the flankers were varied at the same time. Contrast threshold was converted to the log scale, and the average log threshold of the two visual fields for each condition was used. Participants were tested with musical stimuli followed by nonmusical controls. For both types of stimuli, 24 practice trials with feedback were provided before testing (no feedback).

### Measure of perceptual fluency

We assessed fluency in music reading in a sequential-matching paradigm (Wong & Gauthier, 2010a, b). On each trial, a central fixation was presented for 200 ms, followed by a 500-ms premask and a four-note sequence for a varied duration. After a 500-ms postmask, two four-note sequences appeared side by side, one of which was identical to the first sequence, and the other had one of the notes shifted by one step (i.e., a note on a line was moved to an adjacent space, or a note on a space to an adjacent line; shifts were randomly chosen from among the four notes, with up/down shifts counterbalanced). The task was to select the matching sequence via keypress. The presentation duration threshold for 80% accuracy was estimated four times, each time over 40 trials, using QUEST. Sequences were randomly generated using notes ranging from the note below the bottom line (a D note in the treble clef) to the note above the top line (a G note in the treble clef). The contrast for all stimuli was lowered by about 60% to avoid a ceiling effect.

To control for individual differences not specifically tied to expertise with notes, perceptual fluency for four-letter strings was measured by means of an identical procedure. The strings were randomly generated with 11 letters: b, d, f, g, h, j, k, p, q, t, and y. These letters were selected because they contain parts extending upward or downward, similar to musical notation. To create distractor strings, one of the four letters in the string was selected (counterbalanced across stimuli) and replaced by a different letter randomly drawn from the set. The strings were shown at the same lowered contrast as the musical sequences.

### Basic visual functions

To compare the two groups in terms of basic visual functions, far and near acuity values and functional contrast sensitivity were measured (Stereo Optical Vision Tester; Chicago, IL). The acuity test involved reading out uppercase letters presented in different sizes. To measure functional contrast sensitivity, participants judged whether the presented gratings were tilted to the left or the right or were straight up. The gratings were presented with different spatial frequencies (1.5, 3, 6, 12, or 18 cpd) in different contrasts. All of the tests were performed with both eyes and with corrected vision (if needed).

## Results

Four experts were excluded from the data analyses because their perceptual fluency for notes or letters was >3 *SD*s away from the mean of the rest of the group. Therefore, 20 experts and 20 novices were included in the following analyses.

### Crowding

Preliminary analysis of variance (ANOVA) results revealed that experts performed better than novices for the musical baseline condition. To ensure that any expertise effect of crowding was not a result of baseline differences, we further removed the four best-performing experts and the four worst-performing novices in the musical baseline condition.

For staff lines, a 2 × 2 ANOVA with the factors Group (experts/novices) and Crowding (baseline/crowded) on the log contrast threshold revealed a main effect of group, *F*(1, 30) = 4.36, *p =* .045, and a main effect of crowding, *F*(1, 30) = 132.6, *p* < .0001. The Group × Crowding interaction was significant, *F*(1, 30) = 8.78, *p =* .0059 (*η*_p_^2^ = .23, CI_.95_ of *η*_p_^2^ = .02 to .44; Fritz, Morris, & Richler, 2012), with experts performing better than novices only for the crowded condition, but not for the baseline (Scheffé tests, *p <* .05; see Fig. 1).

For flanker notes, a similar ANOVA revealed a marginally significant main effect of group, *F*(1, 30) = 3.94, *p =* .056, and the main effect of crowding was significant, *F*(1, 30) = 178.3, *p* < .0001. The Group × Crowding interaction was also significant, *F*(1, 30) = 8.53, *p =* .0066 (*η*_p_^2^ = .22, CI_.95_ of *η*_p_^2^ = .02 to .44; Fig. 1). Scheffé tests (*p <* .05) revealed that experts performed better than novices for the crowded but not for the baseline condition.

For control stimuli, the same analysis only revealed a main effect of crowding, *F*(1, 30) = 1,408, *p* < .0001. No main effect or interaction involving group reached significance (all *p*s > .2; Fig. 1).

Since performance in the baseline conditions was close to ceiling, potential group differences for baseline conditions might not be able to express, such that the expertise effect for crowding might simply be an artifact. Therefore, we tested the expertise effect for crowding only with the crowded conditions.

For staff lines, the Group × Stimulus (staff lines/Landolt Cs) interaction on log contrast threshold approached significance, *F*(1, 30) = 3.79, *p* = .06. The same analysis for the flanker notes was significant, *F*(1, 30) = 4.37, *p* = .045. In both cases, experts performed better than novices for the musical but not for the control conditions (Scheffé tests, *p* < .05).

In sum, experts experienced less crowding than did novices when the crowding elements were staff lines or flanking notes. However, the amounts of crowding were similar across groups for control stimuli, suggesting that music-reading experience helps alleviate crowding specifically for musical stimuli.

### Perceptual fluency

As expected, experts had higher perceptual fluency than did novices for musical sequences, but not for letter strings. A one-way ANOVA on group (expert/novice) was performed for the perceptual thresholds for matching four-note sequences. Experts required a shorter exposure duration than did novices to match four-note sequences (*M*_Exp_ = 465.5 ms, *M*_Nov_ = 1,281.0 ms; CI_.95_ = 188.1 ms), *F*(1, 30) = 39.2, *p* < .0001, while experts showed no advantage when matching four-letter sequences (*M*_Exp_ = 210.1 ms, *M*_Nov_ = 235.9 ms; CI_.95_ = 60.4 ms, *p* = .5). This suggests that experts have a higher perceptual fluency for reading music sequences, which cannot be explained by a general perceptual advantage.

### Basic visual functions

All of the participants had normal far and near acuity (20/20 or 20/30). All of the participants (except one novice) also had normal functional contrast sensitivity, but excluding the one novice (functional contrast sensitivity = 20/100) did not change the pattern or the significance of the results of the crowding experiment. Therefore, the expertise effect of crowding cannot be explained by differences in basic visual functions.

### Relationship between crowding and reading fluency

We examined the correlations between music-reading fluency, crowding with all three types of stimuli (the contrast threshold difference between the crowded conditions and baseline), and age (Table 1). All of the 20 experts and 20 novices were included. For the fluency measure, we used the note minus letter duration threshold (in millseconds) to minimize individual differences in general perceptual ability. Self-reported numbers of years of music-reading training were not included because this value was zero for the majority of novices, and it was not a significant predictor of music-reading fluency among participants who had a nonzero value in this measure (*N* = 23; *r* = –.234, *p* = .28). One participant was dropped from these analyses because his music-reading fluency was more than 2 *SD*s higher than the mean for the other participants. The results were not qualitatively different when this participant was included.

**Table 1:** Correlations between perceptual fluency in music reading (note – letter); crowding with flanker notes, staff lines, and control stimuli; and age

	Fluency (N–L)	Flanker Notes	Staff Lines	Landolt Cs	Age
Fluency (N–L)	1				
Flanker notes	.518^*^ (.436^*^)	1			
Staff lines	.339^*^ (.308^†^)	.334^*^	1		
Landolt Cs	.166 (.047)	.255	–.081	1	
Age	.473^*^	.330^*^	.151	.266	1

Values shown in parentheses are the correlation values after partialing out the contribution of age in predicting music-reading fluency. Asterisks indicate significant correlations (*p* < .05), and the cross indicates marginal significance (*p* = .06).

**Fig. 3:**
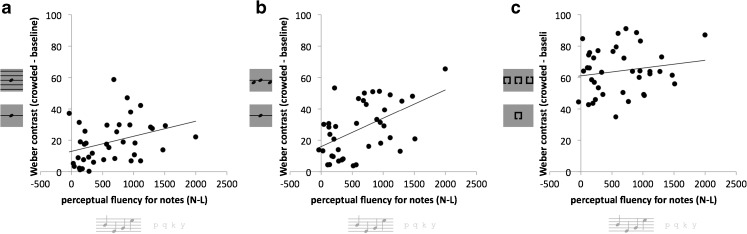
Scatterplots between individual perceptual fluency (notes – letters) and crowding magnitude for (**a**) staff lines, (**b**) flanker notes, and (**c**) control stimuli (Landolt Cs)

For all participants, individual music-reading fluency predicted crowding by staff lines (*r* = .339, *p =* .035; see Fig. 3a and Table 1) and crowding by flanking notes (*r* = .518, *p =* .0007; Fig. 3b), but not crowding with nonmusical controls (*r* = .166, *p* > .3; Fig. 3c). Note that the results remained similar when letter duration threshold was not included as a baseline in the perceptual fluency measure.

Since age was a significant predictor of music-reading fluency (*r* = .473, *p* = .0023; Table 1), we partialed out its contribution and reexamined the correlations between crowding and music-reading fluency. The partial correlations with music-reading fluency were significant for crowding with flanker notes (*r* = .436, *p* = .006; Table 1) and marginally significant for crowding with staff lines (*r* = .308, *p* = .06). However, the correlation was not significant for crowding with control stimuli (*r =* .047, *p* = .78). The partial correlation for age was significant in all three cases (all *p*s < .05). These multiple regressions accounted for 33.6%, 25.9%, and 18.3% of the variance in music-reading fluency, respectively (*R* ^2^ adjusted).

### Musical notation crowding affects discrimination but not detection

To assess whether these effects are related to crowding, we examined a hallmark of crowding: The flanking stimuli that disrupt discrimination should not impair detection of the note (Pelli et al., 2004). Twelve novices performed both the target discrimination task (as described above) and a target detection task, at a given contrast, in which they judged whether the target dot or target Landolt C was present or absent.

**Fig. 4:**
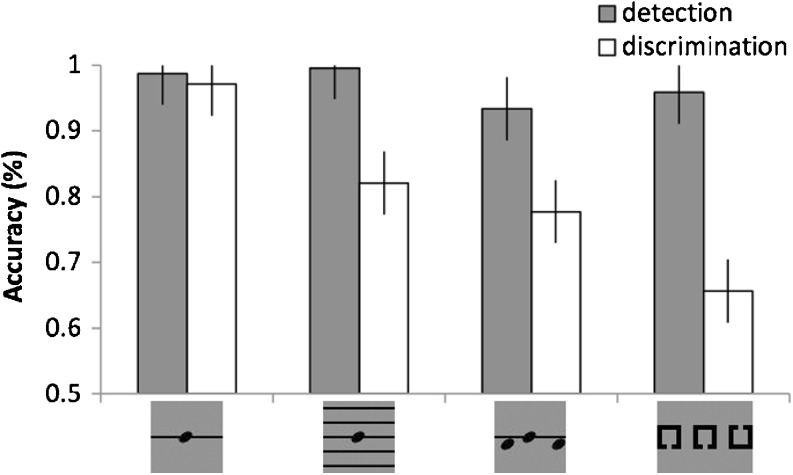
Accuracy for the musical and control stimuli in detection and discrimination tasks. Error bars plot the 95% CIs for the Stimuli × Task interaction, for musical stimuli, or for the main effect of task, for control stimuli

For musical stimuli, a 3 × 2 ANOVA on accuracy with Stimuli (baseline/staff line/flanker notes) and Task (discrimination/detection) as factors revealed a significant interaction, *F*(1, 22) = 6.72, *p* = .005. Scheffé tests (*p* < .05) showed that the detection performance was similar for the three stimuli (Fig. 4). Accuracy for the baseline stimuli was similar for both discrimination and detection, while discrimination was significantly worse with the added staff lines and flanker notes, as compared to the baseline.

For Landolt Cs, accuracy was better for detection than for discrimination in the crowded condition, *F*(1, 11) = 99.9, *p* < .0001 (see Fig. 4). Discrimination for an uncrowded Landolt C was at ceiling (97%; not shown). Detection for an uncrowded Landolt C was not tested, because the screen would have been empty when the only Landolt C was absent.

These results confirm that flanking notes or staff lines affect the discrimination of a note due to crowding (Pelli et al., 2004).

## Discussion

In this study, we tested whether music-reading expertise predicts a reduction in visual crowding and examined how music-reading fluency is related to crowding with musical stimuli. We showed that crowding by flanking notes and staff lines was reduced with music-reading experience. Performance with crowded musical stimuli was predicted by individual music-reading ability. Similar effects were not found with nonmusical stimuli. Since we did not manipulate experience, our results might be explained by selection bias; that is, individuals with better visual spatial resolution *specific to musical notation* are more likely to pursue musical training and to practice music reading. This explanation appears unlikely, however, because both stimulus types are artificial and of no specific evolutionary significance. A more parsimonious explanation is that music-reading training alleviates crowding. These results are consistent with recent evidence about the effect of experience on crowding (Chung, 2007; Green & Bavelier, 2007; Huckauf & Nazir, 2007; Williamson et al., 2009), suggesting that sometimes an improvement in spatial resolution can be specific to a category of objects. Crowding limitations can depend on object category, especially those associated with perceptual expertise, in contrast to a recent proposition that crowding is independent of object categories (Pelli & Tillmann, 2008).

Here, we carefully chose one eccentricity (2.6º) and focused on the crowding magnitude as the first step to demonstrate expertise effects on crowding. Limited by potential ceiling or floor effects, we could not measure crowding at a range of eccentricities. Pilot data revealed that testing at 3º would have led to a floor effect for novices, while testing at 2º would have resulted in a ceiling effect for experts (Fig. 1). In future, other designs should seek to get around such limitations and investigate how perceptual experience affects critical spacing.

For crowding created by staff lines or flanker notes, a smaller crowding effect predicts more fluent music-reading performance. This finding is consistent with theories on word reading that state that crowding limits reading rate (Levi, 2008; Pelli & Tillman, 2008; Pelli et al., 2007). Our results further suggest that the limitations crowding imposes on reading rate are malleable. One possibility is that domain-general individual differences in crowding are small in a novel domain, and their influence may be maximized when assessed in a skilled domain. Future work might compare more than one skilled domain to assess this possibility.

It is worth noting that solving the crowding problem is different from the typical goal of music reading. In crowding, one has to selectively attend to the middle note and ignore the staff lines and flanker notes, while one has to identify all of the notes in normal music-reading circumstances. Prior-training studies have shown that training participants to recognize three-letter trigrams in the visual periphery improves reading rates (Chung, Legge, & Cheung, 2004), while training participants to identify the central, crowded letter in three-letter trigrams did not improve reading rates (Chung, 2007). To speculate, music-reading experience may have specifically improved the visual spatial resolution of musical notation by creating a better representation of the musical elements, including both the staff lines and all of the musical notes. The improved representation of the music sequences would then be useful for the present task, in which selective attention directed to one of the notes was required, and also for achieving a higher fluency for music sequences in general. This may explain why individual differences in crowding could be predicted by fluency of reading in the present study, but not in a previous study involving explicit training on crowding (Chung, 2007).

While improved higher representation of musical notes is a possible mechanism underlying the reduced crowding in experts, other mechanisms are also possible. For example, integration between notes may be reduced with music-reading experience (e.g., Pelli & Tillman, 2008). Also, the resolution of spatial attention may be specifically improved for musical notes as a result of perceptual training (He et al., 1996; Tripathy & Cavanagh, 2002). Although we do not know the exact mechanism(s) underlying the expertise effect on crowding, our results provide important constraints in the theoretical debate of the cause(s) of crowding. Models of crowding need to explain how perceptual experience can lead to a domain-specific reduction of crowding.

Although the neural correlates of crowding remain largely unknown (Levi, 2008), some evidence has suggested that crowding involves multiple visual areas, including V1 to V4 (Fang & He, 2008; Levi, 2008; Millin, Arman, & Tjan, 2010). Recent fMRI evidence has suggested that music-reading expertise is associated with early retinotopic visual cortex showing both increased cortical thickness among musicians (Bermudez, Lerch, Evans, & Zatorre, 2009) and selective increases in activity for single notes in music-reading experts (Wong & Gauthier, 2010b). Bilateral fusiform gyrus has also shown increased selectivity for single notes in experts (Wong & Gauthier, 2010b). The reduced crowding for musical notation may thus involve both early and late areas recruited by music-reading expertise. Furthermore, the visual areas engaged for words and musical notes are distinct (Wong & Gauthier, 2010b). While the reading rates for both words and musical notation may be limited by crowding, further studies should clarify whether crowding in different domains of expertise is determined by activity in different brain regions.
